# Enhancing efficiency in ESD: a comparative analysis of ERBE VIO3 and 300d electrosurgical units

**DOI:** 10.1093/jcag/gwaf011

**Published:** 2025-05-31

**Authors:** Nabeel Ahmed, Mandip Rai, Robert Bechara

**Affiliations:** Faculty of Medicine and Health Sciences, McGill University, Montreal, Canada; Department of Gastroenterology, Kingston Health Sciences Center, Kingston, Canada; Department of Gastroenterology, Kingston Health Sciences Center, Kingston, Canada

## Abstract

**Aims:**

Electrosurgical units (ESUs) are essential for tissue dissection hemostasis during ESD. The ERBE VIO 3, enables rapid setting changes, facilitating the swift application of vessel sealing current. Additionally, features such as PreciseSect mode allow dynamic modulation frequency adjustment, making it suitable for submucosal dissection and vessel management. Our comparison of the ERBE VIO3 and 300d aims to assess whether these functionalities enhance the ESD experience.

**Methods:**

From 2021 to 2024, 88 patients undergoing ESD for colorectal lesions were identified from a prospectively maintained database. Lesions were categorized based on the ESU utilized.

**Results:**

Eighty-eight procedures were identified. Forty-four (50.0%) procedures were performed using VIO 3 and 44 (50.0%) using VIO 300d. 40 (45.5%) lesions were colonic and 48 (54.5%) rectal. Median lesion diameter was 4.5 cm. Lesions in the VIO3 group were significantly larger (*P* = 0.027**).** All ESDs were completed *en bloc*. Use of the VIO3 resulted in a significantly fewer uses of coagulation graspers overall (28 vs 23, *P* < 0.001), fewer uses of coagulation graspers for arterial bleeding (1 vs 2, *P* < 0.001), fewer uses of coagulation graspers per cm^2^ (0.17 vs 0.58, *P* < 0.001), and fewer uses of coagulation graspers per minute (0.011 vs 0.066, *P* < 0.001). This led to a non-significant trend in increased efficiency with use of the VIO3 (4.6 vs 5.1 min/cm^2^, *P* = 0.667).

**Conclusions:**

The VIO 3 significantly decreased reliance on coagulation graspers, particularly in addressing arterial bleeding. This holds the potential to enhance procedural efficiency, reduce bleeding, and lower costs associated with coagulation graspers usage.

## Background and aims

Endoscopic submucosal dissection (ESD) is a safe and effective technique for resecting large superficial colorectal neoplastic lesions en bloc in a minimally invasive fashion.^1^ Vessel management and maintaining hemostasis are critical to the safe and efficient conduct of ESD.

In particular, preventing bleeding is important because stopping bleeding can be challenging and time-consuming, and bleeding in the field can make the tissue layers more difficult to visualize.^2^ Pre-coagulating vessels with electrical current and endoknife compression prior to dissecting them minimizes bleeding. Previous generation electrosurgical units (ESUs) generally have *cut* and *coag* functions for dissection and coagulation, respectively. *Cut* uses a continuous high-intensity current, which vaporizes tissue and results in cutting. *Coag* generally uses pulsed lower intensity current to dessicate the tissue.

When it comes to coagulation, there are soft coagulation modes, which generally use lower voltages, but may not be sufficient if tissue resistance is too high, such as in the case of large vessels. There are also forced coagulation modes, which use higher voltages, however, this can create sparks and cause vessel rupture or thermal injury to deep tissue. Ishida et al. evaluated pre-coagulation with soft coagulation and forced coagulation and found that forced coagulation coagulates tissue wider and deeper than soft coagulation and provides better hemostasis for vessels ≥2 mm in diameter.^2^

If hemostasis cannot be achieved with endoknife compression and applied electrical current, coagulation graspers may be necessary. However, the use of coagulation graspers comes with additional equipment costs and time spent loading them onto the endoscope.

The ERBE VIO3 is a modern electrosurgical unit. In addition to traditional *cut* and *coag* modes, it includes dynamic modes such as PreciseSect, which dynamically adjust energy delivery based on tissue impedance. In comparison, the VIO300d applies a preset current without this dynamic adjustment. This allows for precise tissue dissection while actively sealing small vessels, making it well-suited for submucosal dissection and vessel management. It also facilitates the task of easily switching between various modes via the foot pedal. These dynamic modes and variable current application are advancements compared to predecessor ESUs such as the ERBE VIO300d.

In North America, the implementation of ESD in general is far from ubiquitous. Barriers to the adoption of colorectal ESD in Canada and the United States include training challenges, as well as longer procedure times and a lack of reimbursement.^3,4^ Ensuring that ESD is practiced most efficiently is one way of facilitating the adoption of ESD in North American centers.

To assess whether these functionalities enhance the ESD experience, we compared our experience with ERBE VIO3 and 300d ESUs. We aim to present procedural outcomes with the two units, with a focus on metrics of efficiency and hemostasis.

## Methods

### Study design and patient population

**Figure 1.**
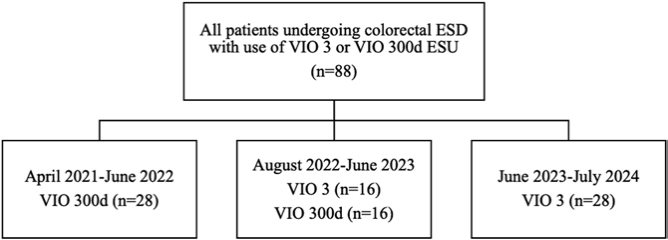
Patient inclusion in the study.

This retrospective, single center case series was performed at a single tertiary-level referral center in Kingston, Ontario, Canada. A study period was selected to include consecutive cases, with equal group sizes, to minimize confounding due to operator experience over time. Between April 2021 and July 2024, all consecutive patients treated with ESD for colorectal lesions were identified from an administrative database, and charts were retrospectively reviewed. Patients who underwent procedures using Erbe VIO 3 and VIO 300d electrosurgical units were identified. 44 patients in each group were identified (Figure 1). Queen’s University Health Sciences & Affiliated Teaching Hospitals Research Ethics Board (HSREB) approval was obtained for the study.

### Variables and data extraction

The primary aim of the study was to compare outcomes from a large single-center case series of colorectal ESD with respect to the electrosurgical unit used in the procedure. Demographic and clinicopathological variables, including ASA score, sex, lesion morphology, procedure time, and lesion size, were extracted from the electronic chart. The procedural variables calculated were efficiency (minutes per cm^2^), lesion area [area of ellipse = π(radius 1)(radius2)], use of coagulation graspers, use of coagulation graspers for arterial bleeding, and use of graspers per unit area. Severe postoperative complications were defined as Clavien Dindo grade ≥2.^5^

### Procedural technique

All cases were performed by one experienced operator (RB) who completed a formal hands-on fellowship at Showa University, Tokyo, Japan. The indications used for ESD in our centre are as follows: JNET 2b, previously resected lesions that were recurrent, rectal lesions that could not otherwise be removed en bloc, and JNET 3 lesions with area less than 1cm, if patients refused surgical resection or wanted it done as a diagnostic ESD prior to surgery.

Procedures were performed under general anesthesia with endotracheal intubation or with conscious sedation. The electrosurgical unit used was determined solely by site availability, with the VIO 300D present at one site but not the other. Endoscopic submucosal dissections were performed at both sites. DualKnife J (KD-655; Olympus) or Flush Knife BTS (DK2620JI; Fujifilm) were used for the procedures. In the VIO 3 group, the first three cases were performed with the DualKnife J 1.5, with the remainder performed using the FlushKnife BTS 1.5. In the VIO 300D group, the first five cases were performed with the DualKnife J 1.5, with the remainder using the FlushKnife BTS 1.5. Precoagulation of vessels with the VIO3 was conducted using *Forced Coagulation* 0.4. Coagulation graspers were not used pre-emptively. Submucosal dissection was then conducted using *PreciseSect* 3.5. With the VIO300d, dissection was conducted using *Swift Coag,* Effect 3, 30-40W. Incision was conducted using *Endocut I*, effect 2-2-2. Coagulation graspers were set to *Soft Coag,* Effect 5, 80W.

The lifting agent was consistent across cases. Hydroxypropyl methylcellulose was used for the initial submucosal injection via an injection catheter. Subsequent injections through the needle utilized Voluven. Underwater ESD was employed throughout for both groups. Traction was not used for any rectal lesions during this time period. However, traction was utilized twice in the colorectal group—once in each ESU group—using clip-and-band traction.

Antithrombotic agents were held in general accordance with American Society for Gastrointestinal Endoscopy recommendations.^6^ The lesions were examined with a combination of white-light, BLI (blue-light imaging), with magnification (up to 135x). JNET classification was assessed by the same operator at the time of ESD or during diagnostic endoscopy. After submucosal injection was performed, mucosal incision was made, and careful dissection of the submucosa was completed. After complete resection and retrieval, the specimens were pinned down and measured, then fixed in formalin for histopathologic examination. ESD was generally performed on an outpatient basis.

### Statistical analysis

The data was processed using SPSS statistical software (version 27, SPSS Inc., Chicago, Illinois, USA). The data is presented as mean or frequency with range or percentage in parentheses. A univariate analysis was performed using Chi-squared or Fisher’s exact test for categorical whilst continuous variables were compared using the Mann-Whitney U test. For all calculations, a *P* value of < .05 was considered statistically significant.

## Results

### Study population

Between April 2021 and July 2024, patients who underwent colorectal ESD with Erbe VIO3 and VIO300d electrosurgical units were identified. Forty-four consecutive patients treated with each ESU were included. Baseline demographic and clinicopathological variables of the study population are described in Table 1. Most of the patients were male (54.5%, 48/88) with a median age of 70 years (range 43-91). Most were ASA 3 (65.9%, 58/88). 40 (45.5%) lesions were colonic and 48 (54.5%) rectal. Most lesions were lateral spreading tumors (LST) (65.9%, 58/88). LST subtypes were granular homogenous (2.3%, 2/88), granular mixed (37.5%, 33/88), non-granular flat (6.8%, 6/88), and non-granular pseudodepressed (19.3%, 17/88). Most (85.2%, 75/88) were classified as JNET IIB. Median lesion diameter was 4.5 cm (range 1.5-13.1 cm). Median lesion area was 13.0 cm^2^ (range 1.2-82.3 cm^2^). Lesions in the VIO3 group were significantly larger (16.6 cm^2^ vs 10.2 cm^2^, *P* =.023). There were no other statistically significant baseline differences between groups.

**Table 1.** Patient and lesion characteristics.

Variable (median/frequency (range/%))	All patients (*n* = 88)	VIO300d (*n* = 44)	VIO3 (*n* = 44)	*P*-value
Sex				.087
Male	48 (54.5%)	28 (63.6%)	20 (45.5%)	
Female	40 (45.5%)	16 (36.4%)	24 (55.5%)	
Age (years)	70 (43-91)	70.5 (43-91)	70 (44-84)	.622
ASA				.431
2	26 (29.5%)	15 (34.1%)	11 (25.0%)	
3	58 (65.9%)	28 (63.6%)	30 (68.2%)	
4	4 (4.5%)	1 (2.3%)	3 (6.8%)	
Non-LST	30 (34.1%)	15 (34.1%)	15 (34.1%)	.184
LST-Granular homogenous	2 (2.3%)	2 (4.5%)	0 (0.0%)	
LST-Granular mixed	33 (37.5%)	13 (29.5%)	20 (45.5%)	
LST-Non-granular flat	6 (6.8%)	5 (11.4%)	1 (2.3%)	
LST-Non-granular pseudodepressed	17 (19.3%)	9 (20.5%)	8 (18.2%)	
JNET classification:				.382
IIA	7 (8.0%)	4 (9.1%)	3 (6.8%)	
IIB	75 (85.2%)	36 (81.8%)	39 (88.6%)	
III	5 (5.7%)	4 (9.1%)	1 (2.3%)	
*n*/a	1 (1.1%)	0 (0.0%)	1 (2.3%)	
Localization				1.000
Colon	40 (45.5%)	20 (45.5%)	20 (45.5%)	
Rectum	48 (54.5%)	24 (54.5%)	24 (54.5%)	
Lesion diameter (cm)	4.5 (1.5-13.1)	4.2 (1.5-10.6)	5.6 (2.3-13.1)	**.027**
Lesion area (cm^2^)	13.0 (1.2-82.3)	10.2 (1.2-68.3)	16.6 (3.5-82.3)	**.023**

### Procedural outcomes

ESD (100.0% (88/88)) were successfully completed, and outcomes are described in Table 2. 100.0% (88/88) were completed en bloc, and 93.2% (82/88) were R0 resections (R1: 1 deep, 4 lateral, 1 peripheral). Median procedure time was 68 minutes (range 15.0-230.0 minutes).

**Table 2.** Procedural outcomes.

Variable (Median/Frequency (range/%))	All patients (*n* = 88)	VIO300d (*n* = 44)	VIO3 (*n* = 44)	*P*-value
Technical success	88 (100.0%)	44 (100.0%)	44 (100.0%)	1.000
En bloc resection	88 (100.0%)	44 (100.0%)	44 (100.0%)	1.000
R0 resection	82 (93.2%)	39 (88.6%)	43 (97.7%)	.091
Procedure time (min)	68.0 (15.0-230.0)	54.5 (11.0-230.0)	91.0 (15.0-360.0)	.218
Coagulation graspers used during the procedure	51 (58.0%)	28 (63.6%)	23 (52.3%)	.280
# of grasper uses	2.5 (0-10)	3.0 (0-21)	1.0 (0-15)	**<.001**
# of grasper uses for arterial bleeding	2 (0-9)	2.0 (0-15)	1.0 (0-10)	**<.001**
# of grasper uses/cm^2^	0.39 (0-6.0)	0.58 (0-6.0)	0.17 (0-3.4)	**<.001**
# of grasper uses/min	0.035 (0-0.23)	0.066 (0-0.23)	0.011 (0-0.13)	**<.001**
Efficiency (min/cm^2^)	4.33 (2.0-10.0)	5.1 (1.7-17.9)	4.6 (2.0-17.1)	.667
Adverse events	1 (1.1%)	0 (0.0%)	1 (2.2%)	.189

Use of the VIO3 ESU compared to VIO300d resulted in a significantly fewer uses of coagulation graspers during procedures (median 28 vs 23, *P* <.001), fewer uses of coagulation graspers for arterial bleeding (median 1 vs 2, *P* <.001), fewer uses of coagulation graspers per cm^2^ (median 0.17 vs 0.58, *P* <.001), and fewer uses of coagulation graspers per minute (median 0.011 vs 0.066, *P* <.001). Although this led to a trend in increased efficiency with use of the VIO3 (median 4.6 vs 5.1 min/cm^2^), this was not statistically significant (*P* =.667). There was a trend towards increased frequency of coagulation grasper use with rectal lesions overall and for arterial bleeding, but this was not statistically significant (*P* =.078 and *P* =.081, respectively). A higher proportion of VIO 3 cases were done under GA (65.9% vs 43.1%, *P* =.032) compared to VIO 300d cases. There was no difference in efficiency between sedation groups (*P* =.519). Severe adverse events occurred in one patient in the VIO3 group, which was a cecal delayed perforation, requiring surgical correction.

## Discussion

In this study, we presented our experience in performing colorectal ESD with ERBE VIO3 and 300d electrosurgical units in a North American setting. We aimed to identify parameters of potentially enhanced procedural efficiency, reduced bleeding, and lowered costs with the use of a modern ESU.

There were 44 patients in each treatment group. The only baseline difference between groups was that lesions in the VIO3 group were significantly larger than those in the 300d group. Eighty-eight resections were attempted and completed. Procedural outcomes were favorable, as 100.0% (88/8) were completed en bloc and 93.2% (82/88) as R0 resections. There was a trend towards improved R0 resection rate in the VIO3 group, however, this was not statistically significant.

Despite lesions in the VIO3 group being larger than those in the VIO300d group, there was no significant increase in procedure time across groups. There was indeed a trend towards improved individual procedural efficiency with the VIO3 unit as well. Although this did not amount to a statistically significant difference in individual efficiency, it may have reached the level of statistical significance in a larger sample size.

Regarding the use of coagulation graspers, procedures performed using the VIO3 required significantly fewer uses of coagulation graspers overall (Median 28 vs 23, *P* < 0.001) and for arterial bleeding (median 1 vs 2, *P* < 0.001), as well as significantly fewer uses of coagulation graspers per minute (median 0.011 vs 0.066, *P* < 0.001) and per cm^2^ (Median 0.17 vs 0.58, *P* < 0.001). This is likely due to the fact that dynamic frequency modulation through modes such as PreciseSect, combined with the clinician’s ability to rapidly switch between modalities—such as low-power forced coagulation—for quick vessel sealing with the endoknife, allows for efficient vessel management without the need to switch to coagulation graspers. There were less procedures requiring the use of coagulation graspers at least once in the VIO3 group (52.3% vs 63.6%), but this difference was not significant (*P* = 0.280). However, when stratified by lesion morphology, among patients with non-LSTs, there was a significant decrease in the number of procedures requiring use of graspers (33.3% vs 80%, *P* = 0.01).

A significant reduction in number of procedures where coagulation graspers were needed, at least among non-LSTs, has the potential to decrease operating costs. Although not significant across all subgroups, this trend may have reached the level of statistical significance with a larger sample size and merits investigation in larger prospective studies. Given that the lesions in the VIO3 group were also significantly larger, if adjusted for size, this may also become significant. Additionally, having to switch to coagulation graspers less frequently subjectively improves the experience for the ESD operator. The significantly fewer uses of coagulation graspers for arterial bleeding may signify improved hemostasis, which may also improve the quality of visualization.

Strengths of this study include the granularity of data afforded by our prospectively maintained database and a relatively large sample size. This study included a fairly comorbid population with relatively large colonic and rectal lesions, which is representative of real-world North American practice. Despite its merits, there are limitations to our study. Although we had a relatively large number of patients undergoing colorectal ESD, the study was likely underpowered to detect differences in some metrics. Additionally, the single-operator and single-center nature of this study may limit the generalizability of the findings to a broader North American population.

Another limitation is the potential confounder of the VIO 3 being used towards the end of the study period, adding experience as a potential confounder for efficiency metrics. In order to avoid this, the study period was selected such that it included consecutive cases, with equal group sizes. In addition, to test for this, only cases August 2022-June 2023 (when both units were used interchangeably) were analyzed separately. The same significant decrease in use of coagulation graspers persisted, as well as trends towards improved efficiency. Opportunities for future research include ongoing development of ESUs and endoknives, which may enable coagulation and dissection with lower voltages.^7,8^

In conclusion, the VIO 3 significantly decreased reliance on coagulation graspers during ESD, particularly in addressing arterial bleeding. This may potentially contribute to enhanced procedural efficiency, reduced bleeding, lower costs, and an improved experience for ESD operators. The value of equipment cannot be understated and innovation in the field of electrosurgery will continue to shape the landscape of therapeutic endoscopy.

## Supplementary Material

gwaf011_Supplementary_Materials

## Data Availability

The data underlying this article will be shared on reasonable request to the corresponding author.
